# Challenges of influencing cellular morphology by morphology engineering techniques and mechanical induced stress on filamentous pellet systems—A critical review

**DOI:** 10.1002/elsc.202000060

**Published:** 2020-11-05

**Authors:** Markus Böl, Kathrin Schrinner, Sebastian Tesche, Rainer Krull

**Affiliations:** ^1^ Institute of Mechanics and Adaptronics Technische Universität Braunschweig Braunschweig Germany; ^2^ Center of Pharmaceutical Engineering (PVZ) Technische Universität Braunschweig Braunschweig Germany; ^3^ Institute of Biochemical Engineering Technische Universität Braunschweig Braunschweig Germany

**Keywords:** filamentous microorganisms, macroparticle, mechanical induced stress, microparticle, morphology engineering, pellet, salt‐enhanced cultivation

## Abstract

Filamentous microorganisms are main producers of organic acids, enzymes, and pharmaceutical agents such as antibiotics and other active pharmaceutical ingredients. With their complex cell morphology, ranging from dispersed mycelia to dense pellets, the cultivation is challenging. In recent years, various techniques for tailor‐made cell morphologies of filamentous microorganisms have been developed to increase product formation and have been summarised under the term *morphology engineering*. These techniques, namely microparticle‐enhanced cultivation, macroparticle‐enhanced cultivation, and alteration of the osmolality of the culture medium by addition of inorganic salts, the salt‐enhanced cultivation, are presented and discussed in this review. These techniques have already proven to be useful and now await further proof‐of‐concept. Furthermore, the mechanical behaviour of individual pellets is of special interest for a general understanding of pellet mechanics and the productivity of biotechnological processes with filamentous microorganisms. Correlating them with substrate uptake and finally with productivity would be a breakthrough not to be underestimated for the comprehensive characterisation of filamentous systems. So far, this research field is under‐represented. First results on filamentous pellet mechanics are discussed and important future aspects, which the filamentous expert community should deal with, will be presented and critically discussed.

**Abbreviations**
CPO: chloroperoxidase
DEM: discrete element method
GFP: Green Fluorescent Protein
MPEC: microparticle‐enhanced cultivation
PBE: population balance equation
VOF: volume of fluid

## 1 INTRODUCTION

Filamentous microorganisms are in the focus of many industrial biotechnological processes for the production of organic acids, enzymes, and pharmaceutical agents such as antibiotics, and are often cultivated as disperse mycelia or pellets in shaken and stirred tank bioreactors [1, 2, 3, 4, 5]. Beside filamentous growing eukaryotic fungi like from the family of *Aspergilli*, also bacterial filamentous microorganisms, actinomycetes, especially *Streptomyces* species, are of great pharmaceutical interest due to their production of various secondary metabolites [6, 7, 8].

**PRACTICAL APPLICATION**

In recent years, a variety of techniques for tailor‐made morphologies of filamentous microorganisms was developed to promote product formation. These morphology engineering techniques range from microparticle‐ and macroparticle‐enhanced cultivation, in which the particles can also have different surface modifications, to salt‐enhanced cultivation by adding inorganic salts (e.g. NaCl, KCl or (NH_4_)_2_SO_4_), to increase broth osmolality and change the cell morphology. The review dicusses how the cell morphology of filamentous microorganisms can lead to higher end product concentrations by these techniques and to what extent micromechanical, multiphase flows and fluid mechanical investigations can support biotechnology in implementing these processes in growth and product formation models, which allow a correlation to productivity. Finally, the remaining gaps and challenges in this complex research area are discussed.

A characteristic feature of filamentous microorganisms is that their growth and productivity are closely linked to particular morphologic phenotypes [9, 10, 11, 12, 13, 14]. The macro morphology determines the micro environment of hyphae through effects on mixing, mass transfer, and broth rheology, which in turn affect product formation [15]. For example, the density of the filament network determines mass transport within a pellet and can be crucial for the viability of filaments inside the pellet and possible substrate limitations. Controlling the morphology of filamentous organisms to increase the yield of a desired product is still a challenging task.

Besides the classical morphology influencing cultivation parameters, e.g. pH value, temperature, culture medium composition, dissolved oxygen, viability/vitality of the inoculum, and inoculum concentration, morphology engineering has been developed in recent years. It is also known that filamentous cell morphology is mainly influenced by mechanical stress. For this reason, the mechanical behaviour of individual pellets is of particular interest for a general understanding of pellet mechanics and the productivity of biotechnological processes with filamentous microorganisms.

This review deals with morphology influencing techniques for filamentous microorganisms and discusses results closely related to cell morphology and pellet mechanics. Therefore, the cell morphological parameters of filamentous microorganisms are presented and discussed in Section 2, followed by the description and evaluation of morphology engineering techniques, i.e. microparticle‐ and macroparticle‐enhanced cultivation and the progress of salt‐enhanced cultivation, which have become part of research in the last 15 years. In Section 3 the experimental investigations for the micromechanical characterisation of pellets are presented. Section 4 discusses the techniques and methods of fluid mechanical models for the description of pellet morphology and their influence in multiphase flows, as they prevail in stirred bioreactors, as well as in shaking reactors with free surface. Section 5 presents the challenges for future research for the understanding of cultivation processes with filamentous microorganisms. The results and findings as well as a look into the future for this highly complex biotechnological topic of filaments in bioprocesses are finally summarised in Section 6.

## 2 CELL MORPHOLOGY OF FILAMENTOUS MICROORGANISMS

One of the most influential properties of filamentous microorganisms is their complex cellular morphology [16, 17, 18, 19]. It is well‐known that there is a strong relationship between the cultivation process conditions, the cell morphology, and the productivity of filamentous microorganisms [15, 20–22]. Thus, the cell morphology is a key aspect in understanding filamentous microorganisms. In submerged cultivations, filamentous microorganisms can develop two different boundary forms of cell morphology depending on the cultivation conditions. In the beginning of filamentous cultivation the differentiation growth process involves different cell types leading from conidia over germ tubes to cylindrical fibers called *hyphae*, which grow and branch. Each individual hyphal filament is surrounded by the cultivation medium and can be described by the micro morphology. Segregation of cells after cell division is not accomplished [18]. Instead, biomass growth is strongly polarised and takes place only at the tip of the hyphae. Tip growth and branching eventually result in different macroscopic appearances (macro morphology) of the culture, ranging from single hyphal elements, so‐called *dispersed mycelia*, over connected networks of hyphae up to distinct spherical particles of biomass termed as (bio)*pellets* [18]. These spherical pellets resemble porous particles, consisting of an outwardly growing densely branched and partially intertwined filament network. In addition to these two boundary forms of cell morphologies, mixed forms can also occur in which the filamentous growing biomass termed as *clumps*. The development of the macro morphology is mainly driven by aggregation, fragmentation, and growth [23, 24]. Dense hyphal networks are known to limit the transport of substrates into filamentous pellets and thus influence their viability and productivity [25, 26, 27].

The availability of growth‐limited substrates (in most cases oxygen) inside a pellet is determined by the equilibrium of the hyphae‐specific oxygen uptake and the convective/diffusive oxygen transport over the pellet surface into the porous hyphal network. The transport processes inside the pellet are mainly influenced by the pellet porosity. The pellet porosity, defined by the density of the hyphal network, describes the free liquid phase between the hyphal structures, in which transport processes can occur. Therefore, the hyphal fraction is often used to characterise the pellet porosity by describing the ratio of the hyphal volume to total volume [25, 28–30]. Hille et al. [25] investigated the hyphal fraction in pellet slices and found a varying porosity over the radius. Further, the authors determined the effective diffusion of oxygen as a function of hyphal density along a radial pellet coordinate. Dense pellets with a large diameter often face oxygen and nutrient limitations in the pellet core [25, 31]. The diffusion of oxygen in the pellet core is hindered when a critical diameter is exceeded. This also influences the microorganisms metabolism and the product formation [32, 33]. However, in very porous or hairy pellets with small diameters the uptake of the growth‐limiting oxygen substrate is not limited in transport.

**FIGURE 1:**
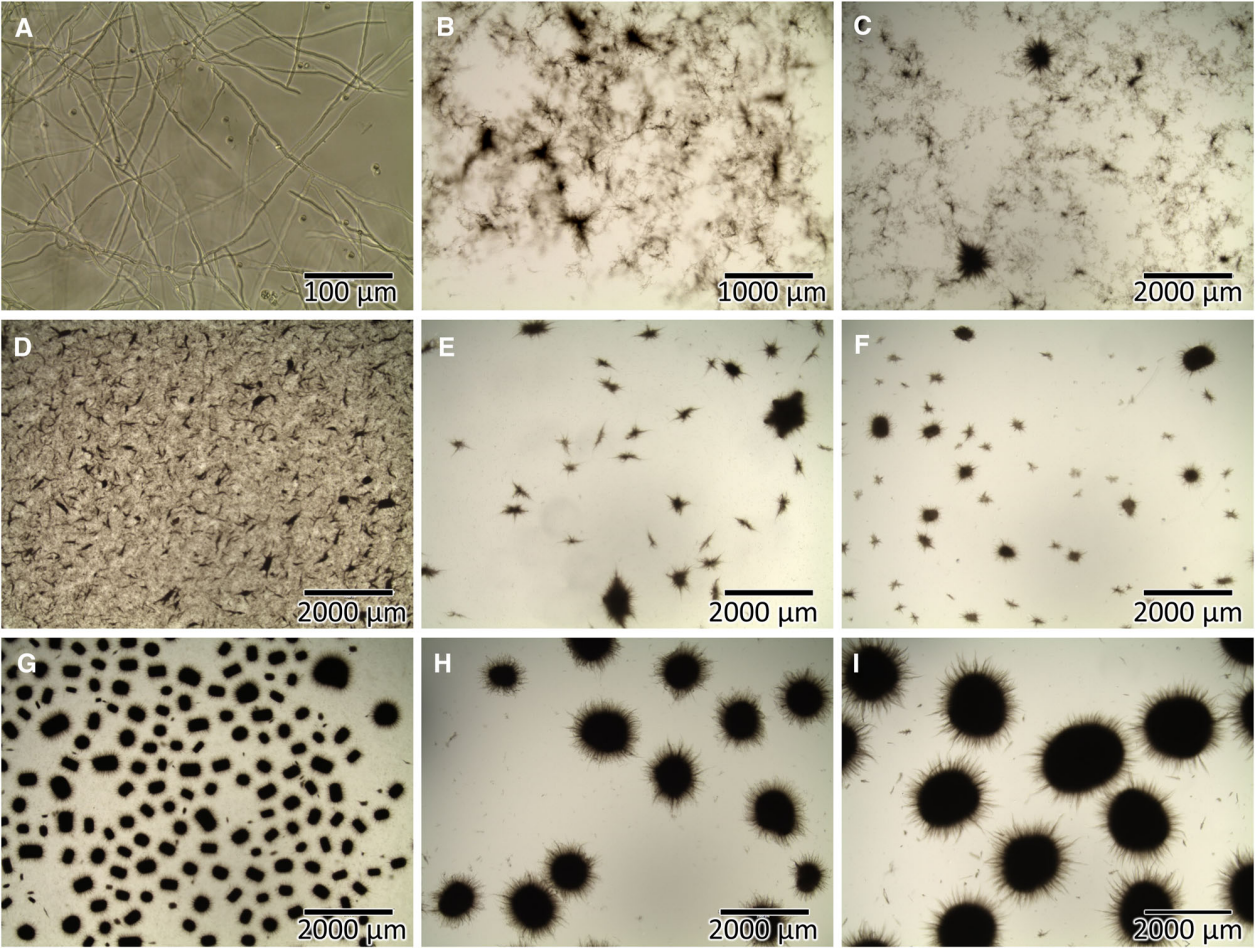
Morphological diversity of the filamentous actinomycete *Lentzea aerocolonigenes* in different cultivations ranging from single hyphae (A), over mostly mycelial structures (B‐D) up to dense and defined pellets in various sizes (E‐I)

The formation of hyphae, which are intertwining to loose mycelia or dense pellets, leads to challenges in the cultivation process [15, 34] as is exemplarily shown in Figure 1.

These challenges are accepted because many filamentous bacteria and fungi produce substances that are interesting for food or pharmaceutical industry [34]. The bacterial filamentous Actinomycetes, including *Actinomadura namibiensis* and *Lentzea aerocolonigenes*, are of special interest, because of the production of mainly pharmaceutically relevant secondary metabolites such as labyrinthopeptin and rebeccamycin, respectively [6, 8, 35]. In 2000, 60% of the known biologically active secondary metabolites were isolated from Actinomycetes [35]. Although there are clear differences between filamentous bacteria and fungi, their morphology and its influence during a cultivation process are similar [6, 36].

In industrial cultivations, mycelium and pellet populations are characterised by different, often contradictory advantages and disadvantages [37]. In comparison to mycelial cultures, pellet cultivations have a lower apparent viscosity and often require a lower power input and lower operating costs for suspension and mixing [38]. Compared to mycelial cultures, pellet‐like growing microorganisms are easier to separate for processing [39, 40, 41].

The different morphological forms of filamentous microorganisms can affect the cultivation process by influencing different parameters such as the viscosity of the cultivation broth and therefore the mixing performance as well as the costs for downstream processing [15]. At high biomass concentrations mycelial structures or pellets with many freely exposed hyphae in their periphery can lead to a high viscosity with a non‐Newtonian flow behaviour [15, 42, 43]. In a highly viscous cultivation broth, the mass transfer and mixing performance are impeded. In order to ensure a sufficient nutrient and oxygen supply of the microorganism an increased power input is necessary [38]. A large power input in turn causes high mechanical stress and therefore leads to morphological changes [44] as well as variations in biomass growth and product titers [45].

For many filamentous microorganisms a correlation between cell morphology and productivity has been observed [46]. Depending on the strain of the microorganism and on the product, the morphological form (mycelium, clumps, pellets) varies, allowing the highest possible productivity in a bioreactor. So far, no strain‐, genus‐family‐, or product‐type related correlation exist to improve process understanding of filamentous production systems [34, 47].

Mycelial structures are advantageous for the production of geldamycin in *S. hygroscopicus* and tylosin in *S. fridae* [48, 49]. The production of citric acid [17, 50], glucose oxidase [44], glucoamylase [29], and polygalacturonidase [51] is favoured in pellet growth by *Aspergillus niger*. Compared to mycelium cultures, pellet morphologies have also been used to achieve higher productivity in the production of nikkomycin by *Streptomyces tendae* [52], avermectin by *Streptomyces avermitilis* [53], rebeccamycin by *Lentzea* (formerly *Lechevalieria*) *aerocolonigenes* [7, 19, 54, 55], and the new peptide antibiotic labyrinthopeptin A1 by *Actinomadura namibiensis* [8]. For these reasons, a large number of studies focus exclusively on pellet‐like cultivations and the interaction between mechanically induced morphological changes and product formation. However, there are also microorganisms that exhibit nearly equal productivity in different morphological forms like *S. clavuligerus* and *S. virginiae* when producing clavulanic acid or virginiamycin [56, 57].

### 2.1 Control and design of the pellet morphology

Cellular morphology, productivity, and the initiation of mycelial or pellet‐like fungal and bacterial growth from an inoculum are influenced by various process conditions, e.g. strain, inoculum size and type, composition of the cultivation medium, pH value, dissolved oxygen content, temperature, presence of certain ions and, volumtric power input and therefore mechanical stress during cultivation [15, 17, 34, 38]. These cultivation parameters of a bioprocess, which influence cellular morphology and productivity, are also summarised under the holistic term *environome* [15, 58]. By adjusting the cell morphology according to the preferences of the microorganism, a targeted optimisation of the product concentration is aimed for. Consequently, various aspects have to be considered.

**FIGURE 2:**
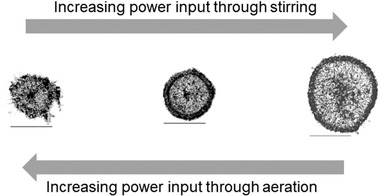
Microscopic images of equatorial pellet sections for the cultivation of *Aspergillus niger* AB1.13 after 62 h of cultivation in a 28 L stirred tank bioreactor with constant volumetric power input P/V of 143 W m^−3^ and non‐limiting oxygen supply at different combinations of the stirrer‐ and aeration‐related power input. Left: P/V 103 (aeration, aer) and 40 (stirring, stir) W m^−3^; middle: 78 (aer) and 65 (stir) W m^−3^; right: 52 (aer) and 91 (stir) W m^−3^; scale bar 500 μm). Adapted from Lin et al. [29]

In pellet cultures, the mechanical interactions of the porous structures with the surrounding turbulent flow, the aeration, the stirrer, and the reactor wall play an important role, as they can cause permanent deformations and rearrangements or densifications of the outer filament network, cf. Figure 2.

Consequently, these morphological changes significantly affect the substrate transport inside the pellet and the filament productivity. For multiple filamentous microorganisms a high power input and high dissolved oxygen levels lead to pellet‐like growth, while a lower power input generates fluffier pellets and low dissolved oxygen levels promote mycelial growth [52, 53, 59, 60]. Besides mechanical stress, the pellet morphology can also be influenced by other cultivation parameters, e.g. substrate supply or cultivation time [52, 61, 62, 63].

#### 2.1.1 Micro‐ and macroparticle‐enhanced cultivation

In recent years, various techniques for tailor‐made morphologies of filamentous microorganisms to promote product formation were developed and summarised under the term morphology engineering [12, 20, 54, 64, 65, 66, 67, 68, 69]. One approach to influence the cell morphology and productivity of filamentous microorganisms is the addition of microparticles, which is meanwhile a frequently used method by researchers [45, 70, 71]. This approach of morphology engineering is called *microparticle‐enhanced cultivation* (MPEC) [63]. The microparticles used in these cases usually have a diameter of <50 μm [54, 67, 72], in some cases even larger particles are treated as microparticles [73, 74].

The first application of microparticle addition to filamentous microorganisms was described by Kaup et al. [64]. The authors investigated the effects of aluminum oxide (Al_2_O_3_) and talc powder (3MgO∙4SiO_2_∙H_2_O) on growth and chloroperoxidase (CPO) formation of *Caldariomyces fumago*. Both types of microparticles had diameters ≤42 μm. The microparticle concentration was varied between 0.05 and 25 g L^‐1^. For an Al_2_O_3_ concentration of 15 g L^‐1^ a 4‐fold increase in accumulated CPO activity was observed after 12 days of cultivation. For 10 g L^‐1^ of talc powder even a 10‐fold increase of accumulated CPO activity was found. Moreover, the cell morphology of the fungus was significantly influenced by the addition of microparticle. Without particles *C. fumago* grew in pellets with a diameter of approximately 4 mm. With Al_2_O_3_ or talc powder the diameter was between 0.1 and 0.5 mm and many single hyphae between 60 and 600 μm were apparent. A similar effect on morphology was also observed for eight other filamentous fungi and the filamentous bacterium *Streptomyces aurefaciens* [64]. The addition of titanate microparticles to *A. niger*, however, resulted in pellets with a dense microparticle core [12].

Since then, many researchers worked with the procedure of MPEC. An overview of different studies applying MPEC is given in Table 1.

**TABLE 1:** Overview of selected applications of MPEC with different microorganisms from the literature

Microorganism	Microparticle type	Microparticle concentration [g L^‐1^]	Reference
*Caldariomyces fumago*	Aluminumoxide, talc	0.05–25	Kaup et al. [63]
*Aspergillus niger*	Aluminum oxide, talc, titanate	0–30	Driouch et al. [12, 72]
*Aspergillus niger, Trichoderma atroviride*	17 particle types	20	Etschmann et al. [73]
*Streptomyces* sp. M‐Z18	Talc	0–20	Ren et al. [75]
*Aspergillus terreus*	Talc	1–15	Gonciarz and Bizukojc [67], Gonciarz et al. [76]
*Aspergillus sojae*	Aluminumoxide, talc	0–25	Karahalil et al. [71], Yatmaz et al. [77], Germec et al. [78]
*Aspergillus niger*	Talc	12	Kowalska et al. [79]
*Lentzea aerocolonigenes*	Talc, surface modified talc	10	Walisko et al. [54]
*Trichoderma viride*	Aluminum oxide	0–30	Dong et al. [80]
*Grifola frondosa*	Talc	0–20	Tao et al. [81]
*Aspergillus terreus*	Aluminum oxide	6–12	Boruta and Bizukojc [82]
*Aspergillus terreus, Penicillium rubens, Chaetomium globosum, Mucor racemosus*	Aluminum oxide	6	Kowalska et al. [83, 84, 85]
*Aspergillus terreus*	Talc	0.48	Saberi et al. [86]
*Aspergillus nidulans*	Talc	20	Niu et al. [74]

As can be seen in Table 1, most MPEC approaches used filamentous fungi, while filamentous bacteria are under‐represented in this field of research. Furthermore, talc and aluminum oxide microparticles are used in almost all studies. Other microparticles were investigated by Etschmann et al. [73]. The effects of 17 microparticle types, differing in size (ø = 5‐250 μm) and chemical composition, were examined in cultivations of a 2‐phenylethanol producing *A. niger* and a 6‐pentyl‐α‐pyrone producing *Trichoderma atroviride*. However, the effects were highly strain‐ and particle‐specific and a general mechanistic description was not possible [73].

Etschmann et al. [73] proposed the design of tailor‐made particles with a defined surface, since the inhomogeneity regarding particle size and shape of different microparticle types might play a major role with respect to their effects on cultivations of filamentous microorganisms. Walisko et al. [54] applied a layer‐by‐layer surface modification technique on talc particles. Thus, talc particles with different surface modifications and thereby different surface properties (e.g. hydrophilic/hydrophobic) were created. These surface modified talc particles were then used in cultivations of *L. aerocolonigenes*. Ten different modifications together with unmodified talc were investigated. The production of rebeccamycin with some modified particles was higher than with unmodified talc, in other cases the product concentrations were lower. Rather hydrophilic or only slightly hydrophobic particles with a negative zeta potential showed the highest rebeccamycin concentrations. However, no clear trend of which properties are advantageous could be identified [54]. Further investigations to the type of surface modifications of particulate additives are required.

The mechanisms behind MPEC are widely discussed, but not yet fully understood. Driouch et al. [65] traced the effects of talc particles on morphology of *A. niger* back to the hindered spore aggregation before germination. Kowalska et al. [85] investigated the addition of aluminum oxide on four fungi with different agglomeration types and considered this aspect to be of major importance in MPEC cultivations. Driouch et al. [65] proposed the loosened mycelial structure, which enables an increased nutrient and oxygen supply to all areas as the reason for the increase in enzyme activity with talc or aluminum oxide. Although the addition of titanate led to a different cell morphology, a loosening of the pellet structure was nevertheless observed, which led to similar improvements in nutrient and oxygen supply throughout the pellet [12]. Talc and titanate microparticles were also added to a cultivation of the recombinant reporter strain *A. niger* ANip7‐MCS‐gfp2, producing a variant of Green Fluorescent Protein (GFP) which was coexpressed with the desired enzyme glucoamylase. While a pellet from an unsupplemented cultivation was only producing in a thin layer on the surface of the pellet, pellets grown with titanate produced GFP all over the pellet [12]. Mycelial structures created by the addition of talc also showed production in all areas [11].

Since the addition of microparticles proved to be beneficial for many filamentous microorganisms, some authors questioned whether larger particles might have similar effects. Kaup et al. [64] tested quarz (SiO_2_) particles with a diameter of approximately 350 μm and glass beads with a diameter of about 500 μm in cultivations of *C. fumago*. The quarz particles showed a qualitatively similar effect as the microparticles for biomass and CPO formation, but much less pronounced. The glass beads, however, did not affect biomass and CPO formation in the cultivation [64]. The addition of broken and porous SiO_2_ particles (ø = 120–200 μm) and glass beads (ø = 250–500 μm) to two different *Streptomyces* strains was investigated by Holtmann et al. [87]. 5 g L^‐1^ of SiO_2_ in a cultivation of *Streptomyces coelicolor* led to an increase in actinorhodin concentration of 85 up to 160% depending on the applied culture medium. The final streptavidin concentration after a cultivation of *Streptomyces avidinii* was not affected by the addition of 5 g L^–1^ glass beads. However, an accelerated production was observed [87].

Neither of the two authors mentioned above investigated the morphological changes caused by the addition of larger particles. Ochi [88] described the homogenisation of biomass, but did not give further details on the pellet size. Hotop et al. [89] added 4 mm glass beads to their pre‐cultures (50 in the first pre‐cultures, 400 in the second pre‐cultures) to reduce the pellet diameter of *Penicillium chrysogenum*. The pellet diameter in the pre‐cultures was reduced to <300 μm, leading to a diameter reduction from 1 to 0.6 mm in the main culture. The final product concentration of penicillin V was doubled [89]. The addition of glass beads was also investigated by Dobson et al. [48]. Beads with a diameter of 5 mm were used in a 250 mL shaking flask for the cultivation of *Streptomyces hygroscopicus var. geldanus*. Between 0 and 55 glass beads were added at the beginning and a spore suspension was used for inoculation. With increasing glass bead number, the average pellet diameter decreased. These changes in cell morphology were accompanied by an increased final geldamycin concentration with increasing glass bead number [48]. Sohoni et al. [90] applied glass beads between 0.75 and 4 mm in microtiter plate cultivations of *S. coelicolor*. Diameters of 0.75 ‐ 2 mm led to pellet‐like growth, while diameters of 3 and 4 mm produced dispersed mycelium. A glass bead diameter of 3 mm was considered optimal, as it led to reproducible and narrow defined morphology of *S. coelicolor* and, in addition, improved the product concentrations of actinorhodin and undecylprodigiosin [90]. The addition of glass beads to *L. aerocolonigenes* was described by Walisko et al. [54]. Beads, featuring diameters between 0.25 and 2.1 mm in a concentration of 80 g L^‐1^, were added to 250 mL baffled shaking flasks. In Walisko [91] even larger particles up to 5 mm were tested in cultivations. With increasing bead diameter, the pellet diameter decreased, resulting in a mycelial structure with glass beads of ≥3 mm glass beads. With 0.25‐0.5 mm beads a 19‐fold increase in rebeccamycin concentration compared to an unsupplemented control was observed [54, 91].

The mechanical stress induced by the glass beads is often considered to be responsible for the effect in cell morphology, since mechanical stress by aeration or agitation can also influence the pellet morphology [29], cf. Figure 2. For a precise estimation of the mechanical stress induced by macroparticles in shaking flask cultivations, simulations of the system are necessary (Section 4).

#### 2.1.2 Salt‐enhanced cultivation

In addition to the techniques of microparticle‐ and macroparticle‐enhanced cultivation, the manipulation of osmolality by adding inorganic salts to the cultivation broth was found to be another method to adapt the cell morphology [66]. In analogy to MPEC this tool of morphology engineering was refered to as *salt‐enhanced cultivation* [8, 92]. Compared to an unsupplemented cultivation the increase in osmolality or osmolarity through the addition of salt leads to significantly higher productivity in some filamentous microorganisms such as *A. niger* for the production of fructofuranosidase or glucoamylase and *Actinomadura namibiensis* for the production of labyrinthopeptin [8, 66]. The pellet size is reduced while the pellet porosity is increased and a stronger mycelial growth occurred which caused this productivity increase. In all cases, the increased osmolality/osmolarity led to more mycelial and clump growth.

The terms *osmolality/osmolarity* refers to the substance quantity of osmotically active ions or particles of solute per kilogram/liter of solvent. The osmolality/osmolarity of cultivation broths mainly depends on the composition of the culture medium and changes during cultivation due to nutrient consumption, release of metabolic products and addition of an acid or base for pH control. Changes in the external osmolality/osmolarity trigger water fluxes along the osmotic gradient and require adaptive processes to counter either swelling or dehydration of the cells. Maintaining the hydrostatic pressure is important as a positive turgor is considered as driving force for cell expansion [93]. Microorganisms have developed mechanisms to adjust the intracellular pressure [94]. Because osmolality/osmolarity changes have the same physicochemical effects in all organisms, there are considerable similarities in their responses to osmotic shifts [95]. When medium osmolality/osmolarity is increased, specialised nontoxic osmolytes (*compatible solutes*) are taken up and/or synthesised in the cytoplasm. These molecules are highly soluble and do not carry a net charge on physiological pH. Exemplary osmoprotectants are glycine betaine, dimethylsulfoniopropionate, carnitine, proline, proline betaine, ectoine, trehalose, and gycosylglycerol [93]. Some halotolerant organisms also accumulate inorganic ions (mainly potassium) to balance the osmotic pressure [96].

Since osmoregulation is inseparable from metabolic regulation [95] and metabolic regulation affects morphology and productivity, the addition of salts to the cultivation medium can be used to manipulate the cell morphology. Studies on the impact of different inorganic salts on the morphology of filamentous microorganisms furthermore showed that different types of ions influence the cell morphology in distinct ways. Pellet‐like growth was enhanced by polycations and suppressed by polyanions, leading to the conclusion that ions also influence the agglomeration behavior of the cell walls [32, 48, 97].

Only a few studies are devoted to the effect of so‐called inert salts such as NaCl or KCl, which are supposed to have no effect on metabolism [98, 99, 100]. Bobowicz‐Lassociska and Grajek [99] increased the protein secretion of washed and filtered mycelia of *A. niger* by the addition of KCl [20]. Furthermore, Fiedurek [98] increased the activity of *A. niger*‐expressed glucose oxidase 2‐fold by adding NaCl to centrifuged mycelia, administering an osmotic shock to the fungus. Wucherpfennig et al. [66] investigated the salt‐enhanced cultivation with two *A. niger* strains. Increased NaCl concentrations led to stronger mycelial growth and an 18‐fold increased productivity of fructofuranosidase and a 4.5‐fold enhanced productivity of glucoamylase in *A. niger* SKAn1015 and AB1.13, respectively. However, the observed changes in productivity might not be due to the change in cell morphology alone. The parameter of osmolality might have influence the fungal physiology, which could in turn affect the cell morphology independently. The increase in the observed productivity was shown to correlate with the active pellet surface area [20, 66]. Table 2 shows an overview of selected applications of the manipulation of osmolality by the addition of inorganic salts to the cultivation broth.

**TABLE 2:** Overview of selected applications of the manipulation of osmolality by the addition of inorganic salts to the cultivation broth–The salt‐enhanced cultivation with different microorganisms

Microorganism	Inorganic salt	Salt concentration [mM]/Osmolality [mosmol/kg]	Reference
*Dendryphiella salina*	NaCl NaCl, NaH_2_CO_3_, Na_2_SO_4_, CaCl_2_, RbCl, KCl, MgCl_2_ NaCl or KCl	0–75/‐ 15/‐ 25/‐	Allaway and Jennings [98]
*Aspergillus niger*	KCl	300–3110/‐	Bobowicz‐Lassociska and Grajek [99]
*Aspergillus niger*	NaCl	400–2800/‐	Fiedurek [100]
*Streptomyces azureus*	Mg^2+^, Ca^2+^, Mn^2+^	0.2/‐	Okba et al. [101]
*Aspergillus niger SKAn1015 and AB1.13*	NaCl	‐/350–2550	Wucherpfennig et al., [66]
*Streptomyces coelicolor*	NaCl	<500, >500/ <1070, >1070	Fuchino et al. [102]
*Actinomadura namibiensis*	NaCl, NH_4_Cl, K_2_SO_4_ (NH_4_)_2_SO_4_	0, 25, 50, 75, 100/‐ 200 (only with (NH_4_)_2_SO_4_)/‐	Tesche et al. [8]

Similar to *A. niger*, Okba et al. [101] investigated the impact on cell morphology of *Streptomyces azureus* (no information about the product) by the addition of Mg^2+^, Ca^2+^ and Mn^2+^. Here, the authors showed that the addition all induced pellet‐like growth. Ca^2+^ supplementation led to smaller pellets than Mg^2+^ supplementation and Mn^2+^ induced pellet formation at much lower levels than Mg^2+^ and Ca^2+^. Fuchino et al. [102] observed that osmotic upshifts of sucrose (1.33 osmol kg^–1^) and NaCl (0.97 osmol kg^–1^) both caused a significant change in the hyphal growth of *Streptomyces coelicolor* (no information about the product). After a 2 to 3 h growth arrest due to the osmotic shock, the developed hyphal tips did not grow again, but several new branches emerged from the lateral hyphal wall. The osmotic shock triggered a reprogramming of the cell polarity and a redistribution of the polar growth sites [102].

Similar observations were made by Tesche et al. [8] for the addition of ammonium sulfate to the filamentous actinomycete *Actinomadura namibiensis*. Here, a salt‐enhanced cultivation was used to increase the production of the pharmaceutically interesting peptide labyrinthopeptin A1 in shaking flask cultures. Among the inorganic salts added to a complex production medium, the addition of 50 mM (NH_4_)_2_SO_4_ resulted in the highest concentration of 325 mg L^–1^ Labyrinthopeptin A1 and increased up to sevenfold compared to the unsupplemented control after 10 days of cultivation. Additionally, the performance of other salts containing NH_4_^+^ and SO_4_^2‐^ (e.g. NH_4_Cl, K_2_SO_4_) was significantly lower than the performance of (NH_4_)_2_SO_4_. The changes in cell morphology of *A. namibiensis* compared to the unsupplemented control were also quantified by image analysis. *A. namibiensis* always developed a heterogeneous cell morphology with pellets and loose mycelia. In contrast to the unsupplemented control, the cell morphology of the (NH_4_)_2_SO_4_‐supplemented cultures was characterised by smaller and more spherical pellets, which were more stable against pellet disintegration in the stationary production phase [8].

## 3 EXPERIMENTAL INVESTIGATIONS FOR THE MICROMECHANICAL CHARACTERISATION OF PELLETS

In the past, extensive experimental investigations for the rheological characterisation of pellet‐based cultivations were carried out [15, 42, 43, 103, 104, 105, 106, 107, 108, 109, 110, 111, 112]. Although it is important to determine the viscous properties of a cultivation medium in order to estimate the power input of a process, it is not possible to conclude on the mechanical stress of an individual pellet or its influence on the pellet morphology without making major system assumptions [92, 113].

**FIGURE 3:**
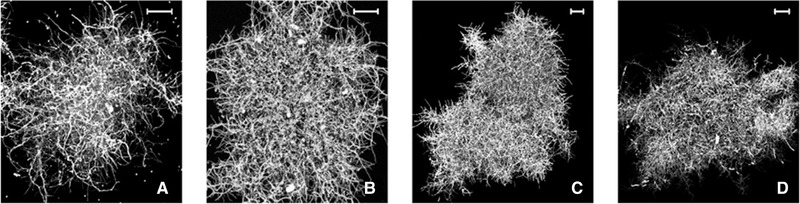
Morphological forms (maximum intensity projections) of pellets and hyphae in a salt‐enhanced cultivation of (A) *A. niger* and 0 mM NaCl, (B) *A. niger* and 500 mM NaCl, (C) *A. namibiensis* and 0 mM (NH_4_)_2_SO_4_, and (D) *A. namibiensis* and 50 mM (NH_4_)_2_SO_4_, recorded by confocal laser scanning microscopy (scale bar 25 μm) [92] (© Copyright 2019 Elsevier B.V.)

So far, the micromechanical behaviour of pellets has mainly been investigated by means of Atomic Force Microscopy (AFM), whereby different growth stages from single hyphae [113, 114, 115, 116] to spores and extracellular matrix adhesion [117, 118, 119, 120, 121, 122, 123, 124, 125] were analysed. Although the mechanical behaviour of individual pellets is of special interest for a general understanding of pellet mechanics, i.e. the understanding of load transfer mechanisms inside the pellet, as well as for biorector and stirrer development and optimisation, only one study exists which deals with the experimental sampling of pellets [92]. In this study, the authors investigate the influence of salt‐enhanced cultivation on the mechanical behaviour filamentous pellets (*Aspergillus niger* and *Actinomadura namibiensis*) by realising cyclic compression experiments. It is well‐known, that pellet growth strongly depends on the cultivation conditions, which was also shown in this study, see Figure 3.

**FIGURE 4:**
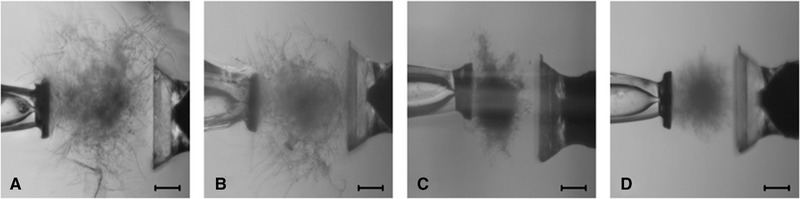
Pellets analysed within Dittmann et al. [92]: (A) *A. niger* and 0 mM NaCl, (B) *A. niger* and 500 mM NaCl, (C) *A. namibiensis* and 0 mM (NH_4_)_2_SO_4_, and (D) *A. namibiensis* and 50 mM (NH_4_)_2_SO_4_ (scale bar: 100 μm) (© Copyright 2019 Elsevier B.V.)

For the realisation of the compression tests, the authors used a custom‐made, micromechanical experimental apparatus, embedded in an inverted microscope. The used setup allowed the measurement of pellets of various sizes and fluffiness, cf. Figure 4. Generally, the pellets showed a nonlinear, dissipative material response with plastic deformations after cyclic loading histories. Further, the use of the strains *A. niger* and *A. namibiensis* under various cultivation conditions led to a similar qualitative, mechanical behaviour. However, clear differences became apparent in the maximum forces and standard deviations achieved for the measurements [92].

These investigations are particularly relevant for the quantification of forces that occur when the pellets collide with the stirrer, the reactor walls or suspended macroparticles and thereby affect the pellet morphology. Consequently, to achieve the highest possible productivity from a process engineering point of view, the mechanical properties of the pellets must lie within the correct process window of mechanical stress. On the one hand, the pellets have to be stable against mechanical loading and on the other hand, they must be open‐pored enough to be fully supplied with oxygen (in case of aerobic cultivation) so that no material restrictions occur. Additional experimental investigations and predictive modelling strategies are necessary in this area.

However, experimental studies at the pellet level are of outstanding importance as they provide key information for a deeper mechanical understanding from a process‐engineering point of view.

## 4 FLUIDMECHANICAL MODELS TO DESCRIBE THE PELLET MORPHOLOGY AND ITS CHANGES

Although the interaction between pellet morphology and mechanical stress is a key essential for the productivity of filamentous biological systems, even after intensive literature research no pellet‐specific models could be found that take into account stress‐induced morphological changes. Many of the existing pellet‐specific models focus on the formation of pellets from single vegetative hyphae or spores and aim to predict the development of inocula in mycelium‐ or pellet‐like cultures [126, 127, 128, 129, 130, 131, 132, 133]. In approaches to predict biomass concentration, substrate uptake, and product formation in ideally mixed bioreactors, pellets are often idealised as spherical, porous particles, growing due to metabolic processes [15, 37]. To this end, a constant density and a radius that changes over time describe the morphology of a pellet. In case substrate limitations occur, the change in radius over time is first approximated by the thickness of the outer active pellet layer in which the nutrient substrates are consumed [6], leading to the so‐called cubic root law, where exponential biomass growth takes place exclusively in the active, substrate‐supplied layer. Cui et al. [134] enhanced the description of a pellet as a porous sphere with a substrate‐limited core by a pellet density that varies linearly with the cultivation time.

**FIGURE 5:**
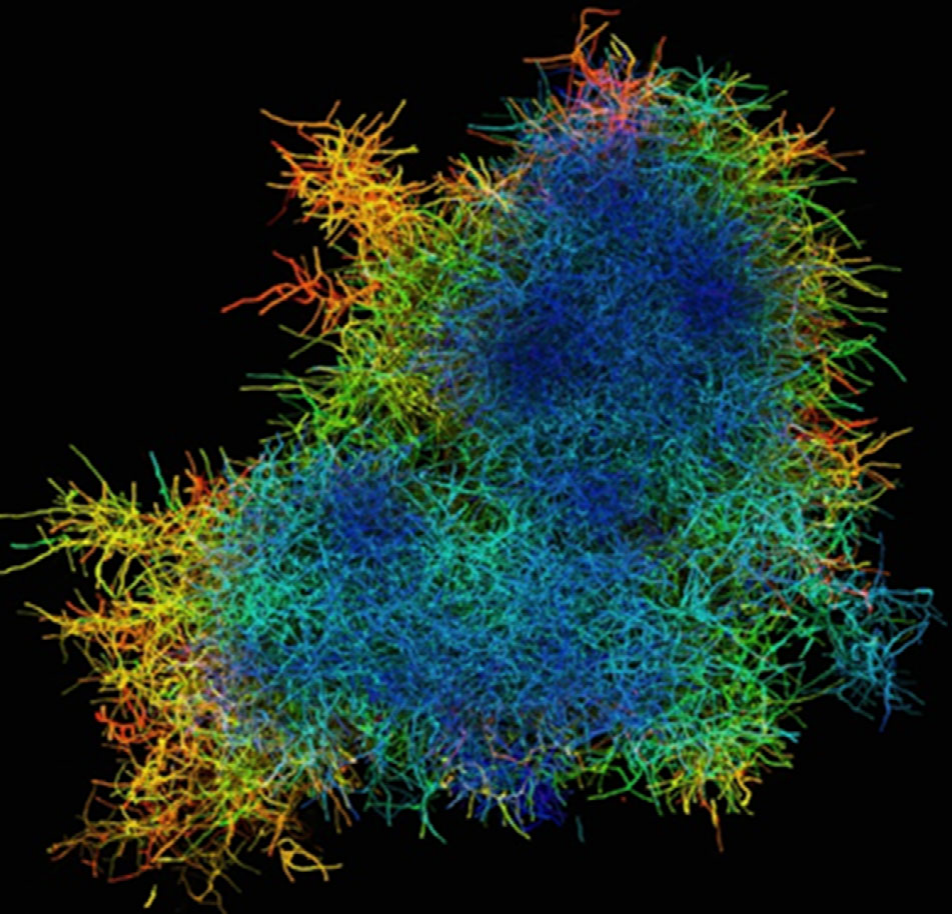
Depth‐coded representation of a filamentous biopellet of *Actinomadura namibiensis*. Note, the colour coding provides information about the position of the hyphae; blue hyphae are close to the microscope objective, red ones are further away

However, in practice, pellets have an almost spherical geometry and a spherically symmetrical density distribution. Morphological investigations on equatorial sections have also shown that the filament distribution and the mass density within a pellet can vary considerably radially [25, 92, 135]. In addition, recent microscopic measurements confirm that the pellet density can vary significantly depending on the radius, see Figure 5. These observations contradict the assumption of a constant pellet density and suggest that spatially inhomogeneous diffusion coefficients and substrate uptake rates need also to be taken into account. Such inhomogeneities influence the pellet internal substrate transport and have a major impact on local substrate limitations and hyphal vitality.

Summarising, new modelling approaches are necessary to resolve these morphological inhomogeneities and their temporal changes and thus to achieve an improved understanding of mechanically induced changes in pellet morphology. These approaches would have to consider the external loads acting on the individual pellet in order to be able to use them independently of a specific reactor configuration or given operating cultivation conditions for the analysis of the pellet morphology distributions and the prediction of product formation.

### 4.1 Multiphase flows in aerobic cultivation

Nutrient media in which pellets are aerobically cultivated are dispersed multiphase systems characterised by intensive coupling at the mechanical and biochemical level. In order to achieve an almost homogeneous dispersion of the pellets and to compensate for gradients of substrate and metabolic products, the cultivation broth is stirred under oxygen supply in industrial stirred tank bioreactors, whereas in bubble column and airlift reactors the mixing takes place exclusively through the pneumatic energy input via the gas phase.

The larger the bioreactor and the more local the energy input for homogenisation of the cultivation broth, the more difficult homogeneous mixing becomes [136]. With a focus on scaling bioreactors from the laboratory scale to industrial sizes, most of the previous research on spatially resolved models has focused on predicting biomass distribution in the bioreactor and productivity for a nominal metabolic kinetics. Mostly planktonic bacterial systems that follow the path lines of the flow and do not collide with each other have been considered [137, 138, 139].

In the case of filamentous pellet cultures, two challenges arise that have not yet been addressed in the literature. On the one hand, pellets grow to diameters larger than 500 μm [92], so that their trajectories deviate from the flow path lines and inertia effects influence the momentum exchange between the pellets and the turbulent flow. On the other hand, the mechanical forces exerted on the pellets by the turbulent flow can cause morphological changes and thus change the productivity of the pellets [17]. The mechanical forces reflect the intermittency of the velocity field to which the pellets are exposed.

The culture medium in stirred or shaken cultures is called *continuous phase* and can be described by the continuity equation and Euler's balance equations for momentum, energy, and mass transport. Depending on the description of the disperse phase, a distinction is made between Euler‐Euler and Euler‐Lagrange approaches. In Euler‐Euler models, the continuous and particulate phases are regarded as interpenetrating continua, each characterised by a volume fraction [140, 141, 142]. Continuity and momentum equations are also formulated for the particulate phase, which can be supplemented by transport equations for the mean properties of the particles, such as mean size. These equations represent the zero and first order moments of a population balance equation (PBE) for the property distribution of the particles at a given position and at a fixed time. The Euler‐Euler method is also known as the two‐fluid method because the continuous and particulate phases behave similarly to two coexisting fluids [143]. Generalis and Cartland Glover [144] used an Euler‐Euler formulation to study the interactions between the gas phase and monodisperse pellets of *A. niger* in a turbulent bubble column reactor for citric acid production. The k‐ε model was used to represent the momentum interactions between the disperse phases and the continuous phase of the liquid culture medium. Elqotbi et al. [145] analysed a laminar stirred reactor with bubble aeration in which *A. niger* produced gluconic acid in mycelium morphology. The mycelium fraction was modelled as a chemical component of the culture medium which causes an increase in viscosity.

A disadvantage of Euler‐Euler formulations is that the basic equations contain mathematical and physical approximations for whose validity and effects our understanding is still limited [146]. Furthermore, Euler‐Euler methods aim at predicting quantities that are averaged over the particle population at a given location. An alternative are Euler‐Lagrange methods in which a separate equation of motion is solved for each particle and the kinetic rates at which the properties of the particles change contribute to changes in the continuous phase. These methods are very well suited for polydisperse particles, whose inertia is variable and whose trajectories can deviate from the path lines of the flow and cross each other [147]. The change in the characteristic particle properties reflects the temporal intermittency of, e.g. resistivity or medium composition. For monodisperse yeast cultures in a stirred tank reactor an Euler‐Lagrange formulation of Lapin et al. [137] was presented and coupled with a detailed metabolic mechanism for glycolysis. In a follow‐up article [138] the authors adapted this formulation to culture broths of *Escherichia coli*, whose intracellular metabolism is controlled by glucose uptake.

A challenge in Euler‐Lagrange approaches for turbulent flows is to reconstruct the instantaneous flow velocity at the position of a particle from the Reynolds‐averaged velocity. For this purpose, the mean flow velocity is extended by a fluctuating component derived from the vortex viscosity [148]. The extended equations of motion represent a stochastic process, the realisations of which correspond to samples from the averaged property distribution of the particulate phase at a certain position. The property distribution can be described by an averaged PBE and the stochastic equations of motion of the particles correspond to a statistically equivalent reformulation of the PBE [149].

Even after intensive literature research, it has been shown that in the existing models for predicting the productivity of pellet cultures, the pellet morphology is assumed to be constant and the influence of morphological changes and the associated metabolic and mechanical changes are excluded.

### 4.2 Modelling of shaking reactors—influence of free surfaces

In shaking flask reactors, oxygen is usually introduced exclusively via the free surface. The shape of the free surface changes over time and influences the shape and position of the flow area in which the pellets are dispersed. Free surfaces exert a dissipative influence on turbulent flows, which dampens the fluctuations in the velocity field [150]. Previous studies on flow conditions and flow properties in shaking flasks used a Volume Of Fluid (VOF) formulation combined with a strategy to reconstruct the free surface between gas and liquid [151, 152, 153]. The VOF method is based on the idea to extend the area for physical modelling to the whole shaking flask and to indicate the phase at a certain location by an indicator field, which varies between 0 (liquid) and 1 (gas) and is transported with the current velocity field. At the interface between the two phases, the indicator field assumes intermediate values which can be used to approximately reconstruct the shape of the interface. Zhang et al. [151] used a VOF formulation to investigate the oxygen transport from the gas phase into the culture liquid of a shaking flask without flow baffles and to quantify the specific power input. This approach was later supplemented by the RNG k‐ε‐turbulence model [150] and applied in a recently published paper [154]. Liu et al. [153] derived a threshold value for the fluid mechanical shear stresses by means of VOF simulation, above which the specific growth rate of plant cells in a shaking flask decreases. In experimental investigations on pellet cultures of filamentous bacteria (*L. aerocolonigenes*) it was observed that the addition of macroscopic glass spheres in shaking flasks can increase product synthesis many times over [54]. One possible explanation is that the pellets are structurally altered in collisions with glass spheres or under the influence of the velocity field altered by the glass spheres, thereby increasing their porosity. This in turn facilitates the transport of oxygen within the pellets and leads to an intensification of the metabolism inside the pellets. To support this hypothesis, Schrader et al. [55] used a VOF‐DEM model to analyse the mean kinetic energy that can be transmitted by dispersed glass spheres in collisions and derived a measure for the stress energy in the dispersion. In the case of fixed mass occupancy, the stress energy increases with the size of the glass sphere. Even though the investigations here show that an optimal stress energy exists for which the pellets are porous without fragmenting, and pellet productivity is highest, the exact mechanism of pellet loosening remains unexplained, except for conjecture.

However, common feature of the above mentioned VOF‐based models is that the dispersed pellets were not represented and the mean energy dissipation rate in the flow [153] or the total stress energy [53] was interpreted as a direct measure of the pellet loads. On the one hand, the intermittency of the loads was not considered. On the other hand, the forces exerted by the flow on the pellets change very much with the size and morphology of the pellets.

## 5 CHALLENGES FOR FUTURE RESEARCH WITH FILAMENTOUS MICROORGANISMS

The huge efforts in quantitative characterisation and tailoring of filamentous cell morphology have shown an evident interrelationship between the operating environment of the bioprocess, productivity, and metabolic properties of the individual bio‐agglomerate (mycelium, clump, and pellet). Nevertheless, the reproducibilities in product titers or cell morphologies to be optimally adjusted via morphology engineering techniques are still very unsatisfactory when cultivating filamentous microorganisms. Closing the gap between the intregral metabolic parameters, small‐scale processes at the pellet level, and the interconnections and transformation across the scales up to the process level remains the most interesting aspect for future research work.

Many existing modelling approaches for describing and predicting the viability and productivity of filamentous pellet cultures are linked to a specific bioreactor system and defined operating and cultivation conditions. Thus, in practice, reaction kinetics are almost without exception averaged over the entire heterogeneous pellet population, even if the individual pellets have very different cell morphologies and thus contribute differently to overall productivity. Therefore, the aim of future research activities in this field should be to develop a transferable, strain‐, genus‐family‐, or product‐type related models to describe the productivity of disperse pellet cultures and to predict process‐relevant properties [30, 43]. For this purpose, integral observation variables such as the mean oxygen‐, biomass‐, or product‐concentration can be traced back to physical processes taking place at the pellet level. The focus on small‐scale processes will make it possible to predict pellet growth, morphological characteristics, and production behaviour for any reactor geometry and operating conditions. In order to bridge the scales between pellet and the bioreactor, a turbulent flow model can be combined with a point particle‐method. The properties of the pellets change due to mechanical and chemical interactions with the surrounding culture medium. By means of special experimental investigations, kinetic rate expressions for these interactions can then be derived, calibrated, and validated.

Still this field is far from understanding the underlying metabolic and regulatory mechanisms. Experimental and computational technologies in systems biotechnology; however, will help to step towards a better understanding of this complex link between the biological and engineering aspects of filamentous microorganisms.

## 6 CONCLUSION AND FUTURE PERSPECTIVES

Due to their ability to produce a wide variety of bioactive substances, many eukaryotic and prokaryotic filamentous microorganisms (e.g. fungi and actinomycetes) are of great interest to the biotech‐related industry. The cell morphology of filamentous microorganisms plays a major role regarding their productivity and can be influenced in different ways. Beside parameters such as pH value, temperature, cultivation medium composition, dissolved oxygen, viability/vitality of the inoculum, inoculum concentration, power input, and mechanical stress, the most important proven technology of morphology engineering is the addition of micro‐ and macroparticles, the microparticle‐ and macroparticle‐enhanced cultivation, and the change of osmolality of the cultivation medium by adding inorganics salts, the salt‐enhanced cultivation. These techniques have already proven useful and now await further proof‐of‐concept ‐ a promising starting point for the next level of filaments in bioprocesses technology.

The mechanical behaviour of individual pellets is of special interest for a general understanding of pellet mechanics. Correlating it with the substrate uptake and finally to the productivity of the filamentous system would be a breakthrough for the comprehensive characterisation of filamentous systems which should not be underestimated. Up to now, this research field is under‐represented. Such investigations are particularly relevant for the quantification of forces that occur when the pellets interact with reactor internals (e.g. stirrer, baffles, reactor wall) or suspended particles and thereby affect the pellet morphology. Additionally, experimental studies at the pellet level are of high importance as they provide key information for the productivity of filamentous biological systems and a deeper mechanical understanding from a process‐engineering point of view.

Summarising, new experimental and modelling approaches are necessary to resolve morphological inhomogeneities and their temporal changes and thus to achieve an improved understanding of mechanically induced changes in pellet morphology. Such approaches would have to consider the external loads acting on the individual pellet in order to be able to use them for the analysis of the pellet morphology distributions and the prediction of product formation independently of a specific reactor configuration or given operating cultivation conditions. In addition it has been shown that in the existing models for predicting the productivity of pellet cultures, the pellet morphology is assumed to be constant and the influence of morphological changes and the associated metabolic and mechanical changes are excluded. Here, additional experimental investigations and predictive modelling strategies are necessary.

## CONFLICT OF INTEREST

The authors have declared no conflicts of interest.

## Data Availability

Data sharing not applicable to this article as no datasets were generated or analyzed during the current study.
